# Characterisation of Early Mucosal and Neuronal Lesions Following *Shigella flexneri* Infection in Human Colon

**DOI:** 10.1371/journal.pone.0004713

**Published:** 2009-03-05

**Authors:** Emmanuel Coron, Mathurin Flamant, Philippe Aubert, Thilo Wedel, Thierry Pedron, Eric Letessier, Jean P. Galmiche, Philippe J. Sansonetti, Michel Neunlist

**Affiliations:** 1 INSERM, U913, Nantes, France; 2 Université de Nantes, Faculté de Médecine, Nantes, France; 3 CHU Nantes, Hôtel Dieu, Institut des Maladies de l'Appareil Digestif, Nantes, France; 4 Institute of Anatomy, University of Kiel, Kiel, Germany; 5 Institut Pasteur, Unité de Pathogénie Microbienne Moléculaire & Unité INSERM 786, Paris, France; Charité, Campus Benjamin Franklin, Germany

## Abstract

**Background:**

*Shigella*, an enteroinvasive bacteria induces a major inflammatory response responsible for acute rectocolitis in humans. However, early effect of *Shigella flexneri (S. flexneri)* infection upon the human mucosa and its microenvironement, in particular the enteric nervous system, remains currently unknown. Therefore, in this study, we sought to characterize *ex vivo* the early events of shigellosis in a model of human colonic explants. In particular, we aimed at identifying factors produced by *S. flexneri* and responsible for the lesions of the barrier. We also aimed at determining the putative lesions of the enteric nervous system induced by *S. flexneri*.

**Methodology/Principal Findings:**

We first showed that, following 3 h of infection, the invasive but not the non-invasive strain of *S. flexneri* induced significant desquamation of the intestinal epithelial barrier and a reduction of epithelial height. These changes were significantly reduced following infection with SepA deficient *S. flexneri* strains. Secondly, *S. flexneri* induced rapid neuronal morphological alterations suggestive of cell death in enteric submucosal neurones. These alterations were associated with a significant increase in the proportion of vasoactive intestinal peptide (VIP) immunoreactive (IR) neurons but not in total VIP levels. The NMDA receptor antagonist MK-801 blocked neuronal morphological changes induced by *S. flexneri*, but not the increase in the proportion of VIP-IR.

**Conclusions/Significance:**

This human explant model can be used to gain better insight into the early pathogenic events following *S. flexneri* infection and the mechanisms involved.

## Introduction

Diarrheal diseases induced by bacterial, viral or parasitic pathogens represent a major world-wide public health problem. Although mortality from diarrheal diseases has been reduced by about 50% over the past 20 years, they still account for 2.5 millions deaths/year [1]. Species of the genus *Shigella flexneri* (*S. flexneri*) are among the most frequently isolated pathogens from patients with diarrhoea [2]. *S. flexneri*, one of the 4 species of *Shigella* is the hyperendemic species in the most impoverished areas, responsible for 10% of all diarrhoeal episodes in children younger than 5 years [3].

The pathogenesis of shigellosis is a multi-step process that has been extensively studied over the past years mainly in animal models or *in vitro*. Schematically, after ingestion, *S. flexneri* colonizes the colon, translocates through the intestinal barrier via M cells, and causes severe destruction of the intestinal epithelium in response to a major intestinal inflammatory response [4], [5]. *S. flexneri* triggers an acute inflammation in part due 1) to apoptosis of monocytes and macrophages, which widely release pro-inflammatory cytokines and 2) to the stimulation of the innate immune response via the activation of Nod- and Toll-like receptors by bacterial cell components [6]. This inflammation facilitates the intestinal barrier rupture and bacterial spread within intestinal epithelial cells at a distance from the initial entry site, further amplifying the inflammatory response and mucosal damage [6]. Although the mechanisms and pathways of infection of *S. flexneri* have been largely identified in animal models [7]–[9], few precise data are available in humans, who are the only naturals host for the bacteria, especially at early times of infection.

Entry of *S. flexneri* in host cells is mediated by the type III secretory system (TTSS) which allows direct activation of components of the cytoskeleton by delivery of dedicated bacterial factors [4]. Other proteins secreted by *S. flexneri* could also be involved in these early events. Among them, the serine protease SepA participates in the pathogenic events induced by *S. flexneri* [10]. Indeed, infection of rabbit ileal loops by *S. flexneri* SepA mutant reduced mucosal atrophy and tissue inflammation induced by the wild type [11]. However, the role of SepA in the effects of *S. flexneri* in the human colon remains currently unknown.

Alterations of the enteric nervous system (ENS) may be involved in the course of *S. flexneri* infection, thus participating in both acute (from mild watery diarrhoea to severe dysentery) and late symptoms. In particular, shigellosis increases the risk to develop post-infectious like symptoms such as irritable bowel syndrome [12], [13]. The ENS, an integrated neuronal network present all along the gut, controls major gastrointestinal functions [14]. In particular, submucosal neurones and glial cells, which densely innervate the mucosa, are key components controlling intestinal barrier functions such as paracellular permeability or intestinal epithelial cell proliferation [15]. In pathological conditions, such as intestinal inflammation, gastrointestinal dysfunctions are associated with changes in the neurochemical phenotype or degenerative process in the ENS [16]. However, the effects of *S. flexneri* upon the phenotype and survival of enteric neurons remain totally unknown, especially in human tissues.

Therefore, the goals of this study were first to characterize *ex vivo* the early events of shigellosis in a model of human colonic tissue. Second, we aimed to identify factors produced by *S. flexneri* and responsible for the lesions of the barrier. In particular, we focussed onto the role of SepA, a factor non dependent of the type III secretion apparatus. Finally, we aimed to characterize the putative lesions of the ENS induced by *S. flexneri* in this human model.

## Materials and Methods

### *Shigella flexneri* (*S. flexneri*)

Four strains of *S. flexneri* were used in this study: 1) the wild type *S. flexneri* 5a (M90T) that harbors a virulence plasmid encoding its invasive phenotype, thereafter called “M90T”, 2) the plasmid-cured mutant which is non-invasive, thereafter referred as “BS176”, 3) M90T deleted for sepA, a plasmid gene encoding for a major serine protease secreted by *S. flexneri*, thereafter called “SepA−“ and 4) BS176 expressing SepA, thereafter referred as “SepA+”. These four strains were stored at +4°C on Petri dishes containing 30 ml of Tryptic Soy Agar (Difco, Becton Dickinson) and Congo red at a concentration of 0.01% (Merck, Darmstadt). Eighteen hours before the experiment, one colony was resuspended in 10 ml of tryptic soy broth and cultured overnight at 37°C. Before tissue infection, bacteria were adjusted to 10^9^cfu/ml by OD measurement (600 nm) and diluted in fresh broth for 2 h in order to harvest bacteria in the exponential growth phase.

### *Ex vivo* human colonic preparations

#### Organotypic culture model

Tissue specimens were obtained from 30 patients (mean age 61 years (21–91)) undergoing surgical resection for colonic adenocarcinoma. None of the patients had bowel obstruction or other colon disease. Specimens were taken at a distance from the tumor in macroscopically and histologically normal areas and immediately processed in the Pathology Department. According to the guidelines of the French Ethics Committee for Research on Human Tissues, these specimens were considered “residual tissues”, not relevant to pathological diagnosis. All specimens were collected prior August 2007. At that time, the specimens considered as “residual tissue” were not subjected to patient consent.

Tissues were placed in oxygenated sterile Krebs solution containing (in mM: 117 NaCl, 4.7 KCl, 1.2 MgCl_2_ 6H_2_O, 1.2 NaH_2_PO_4_, 25 NaHCO_3_, 2.5 CaCl_2_ 2H_2_O and 11 glucose) and then rapidly transported to the laboratory for experiments. The specimen was pinned flat with the mucosa down in a dissection dish containing ice-cold sterile oxygenated Krebs solution which was changed every 10–15 min. Tissue preparations taken from intertaenial regions were 4–7 cm long in circumferential direction, and 4–5 cm long in longitudinal direction.

The serosa and circular muscle were removed under a dissection microscope. The mucosa and submucosa fragments were directly cultured in a Petri dish in the presence of the different strains of *S. flexneri*. The culture medium (Dulbecco's modified Eagle's medium/F12; Sigma; St Quentin Fallavier, France) was supplemented with 10% heat-inactivated fetal calf serum, glutamine (all from Sigma) and 2.1 g.l^−1^ NaHCO_3_. Four different strains of *S. flexneri* (M90T, BS176, SepA− and SepA+) or no bacteria (control tissues) were added to the culture medium. The fragments were maintained for 3 h at 37°C in a humidified incubator containing 5% CO_2_ and air, on a rocking tray. At the end of the experiments, supernatants were removed and immediately stored at −80°C.

### Transmission electron microscopy

For transmission electron microscopy, small sized samples (about 7 mm border length) of the mucosal-submucosal tissue layer were immediately fixed by immersion in 0.1 M cacodylate buffer containing 2.5% glutaraldehyde and 2% paraformaldehyde at pH 7.4 for 24 h. Specimens were post-fixed in 1% OsO4 and stained ‘en bloc’ with 2% uranylacetate. After dehydration in graded alcohol concentrations, the specimens were embedded in araldite. Semi-thin sections were stained with methylene blue and azure II to visualize the regions of interest, in particular the enterocytes lining and the lamina propria mucosae. Ultrathin sections were cut and stained with lead citrate and examined with a transmission electron microscope (Philips, EM109). The images were recorded both by conventional films (Agfa) and digital image system (Soft Imaging Systems, Münster, Germany).

### Immunohistochemistry

Fragments were fixed for 4–6 h in 0.1 M phosphate-buffered saline (PBS) containing 4% paraformaldehyde at room temperature. Following fixation, tissues were washed in PBS and pinned in a dissection dish. The mucosa was then removed under a dissection microscope and the submucosal plexus of Meissner (located directly under the mucosa) was carefully dissected with fine scissors.

### Immunohistochemical staining and analysis of the human colonic mucosa

The mucosa was dehydrated and embedded in paraffin. Sequential sections of mucosa (5 µm in thickness) were made. Infected tissues were observed by classical microscopy following hematoxylin-eosin staining. The length of the epithelial desquamation and the height of the surface epithelium were measured in 10 fields examined under an Olympus IX 50 fluorescence microscope. Pictures were acquired with a black and white video camera (Mod. 4910, Cohu Inc., SL Microtest, Jena, Germany) connected to a Macintosh computer through a frame-grabber card (Scion Image, SL Microtest).

### Immunohistochemical staining of the human submucosal plexus

The submucosal plexus was then permeabilised for 1 h in PBS/NaN_3_ containing 0.5% Triton X-100 (Sigma) and 4% horse serum (Sigma), before being incubated with the primary antibodies i.e., mouse anti-VIP (1∶1000; Biogenesis, UK) diluted in PBS/NaN_3_, 4% horse serum, and 0.5% Triton-X overnight at room temperature. Following incubation with primary antisera, the tissue was washed three times with PBS and incubated for 3 h with secondary antibodies coupled to donkey anti-mouse IgG conjugated to 7-amino-4-methyl-coumarin-3-acetate (AMCA) (1∶50; Euromedex, Mundolsheim, France). In the next step, the tissue was labelled with rabbit anti-neurone specific enolase (NSE) (1∶2000; Biovalley; Marne La Vallée, France) overnight at room temperature. Following incubation with the primary antisera, the tissue was washed with PBS and incubated for 3 h with donkey anti-rabbit IgG conjugated to fluorescein isothiocyanate (FITC) (1∶200 Immunotech, Marseille, France). Specimens were viewed under an Olympus IX 50 fluorescence microscope fitted with adequate filter cubes. Pictures were acquired with a black and white video camera (Mod. 4910, Cohu Inc., SL Microtest, Jena, Germany) connected to a Macintosh computer through a frame-grabber card (Scion Image, SL Microtest).

### Identification of neuronal cell populations

The immunohistochemical study was performed by analysing an average of 25 submucosal ganglia in each preparation. The proportions of neurochemically-identified populations are expressed relatively to the number of NSE-positive neurones per ganglion, which was set to represent 100% of neurones. For the quantitative analysis of NSE immunoreactivity, fluorescence intensity of at least 20–30 submucosal neurons was analysed in each preparation following a method similar to the one previously described [17]. In brief, the outline of the neuron was defined with the cursor and mean fluorescence level of the pixels within the delimited area was determined using Scion Image software program. The background fluorescence of the area next to each ganglia was subtracted from the fluorescence level of each neuron.

### Interleukin-8 and VIP measurements

Supernatants of tissue cultures were collected and IL-8 protein levels were determined by ELISA (B&D, France) according to the manufacturer's instructions. Each sample was assessed in duplicate. For determination of VIP concentration in the submucosal plexus, the proteins of the submucosa were extracted using RIPA lysis buffer (Millipore, Saint Quentin en Yvelines, France) containing protease inhibitor cocktail (Roche Diagnostics, Meylan, France) and levels of VIP were measured by ELISA (Bachem, Weil am Rhein, Germany).

### Pharmacological study

MK-801(Sigma) (50 µM) was applied at least 30 minutes prior to addition of *S. flexneri*.

### Statistical methods

Each single experiment comparing at least two conditions was performed on tissues obtained from the same subject. Each set of experiments was repeated at least five times. Data are expressed as mean±SEM when normally distributed, or as the median (25%–75% percentiles) when non-normally distributed. A paired or unpaired t-test, a Mann-Whitney test, an one-way analysis of variance (ANOVA) followed by a Bonferroni t-test, were performed to compare different populations. Differences were considered as significant for p<0.05.

## Results

### 1. Early effects of *S. flexneri* on human colonic epithelium

#### 1.1 Epithelial alterations induced by *S. flexneri*

**Figure 1:**
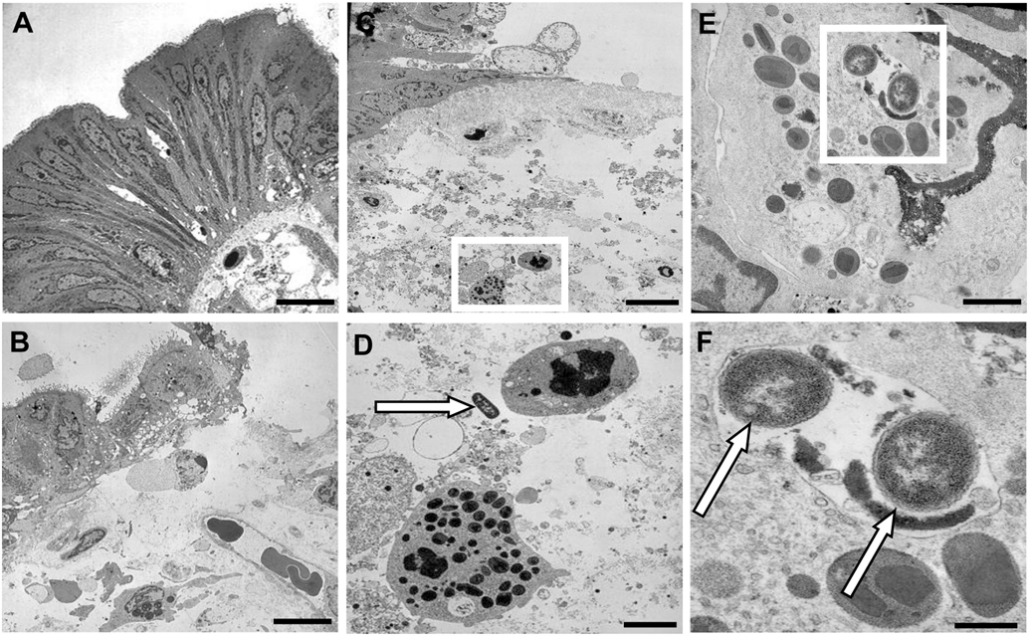
Transmission electronic microscopy of human colonic mucosa infected or not with *S. flexneri* (M90T). (A) Control tissue shows normal epithelial lining with regular basolateral folds and absence of bacteria in the lamina propria. (B) Tissue infected with M90T shows regions of partially detached and vacuolated epithelial cells or regions with complete detachement of epithelial lining. (C) Tissue infected with M90T showing regions of detached epithelial cells and presence of bacteria within the lamina propria adjacent to granulocytes (white square) (D). Magnified view of the selected area from C showing bacteria (arrow) close to granulocytes. (E) Granulocyte containing an intracellular vacuole filled with bacteria (white square). (F) Magnified view of the selected area from E showing two intracellular bacteria (arrows). Bar: 10 µm (A, B, C), 2 µm (D), 1 µm (E), 0.5 µm (F).

We first examined the early alterations induced by *S. flexneri* on the human colonic epithelium using transmission electronic microscopy (n = 5). Control tissues showed normal epithelial lining with normal basolateral folds and without bacteria in the lamina propria (Figure 1A). In contrast, tissues incubated for 3 hours in the presence of the invasive strain of *S. flexneri* (M90T) showed areas of detached and vacuolated epithelial cells and also regions of complete detachement of the epithelial lining (Figure 1B,C). No bacteria was observed inside epithelial cells but in contrast, bacteria could be clearly visible beyond the epithelial lining, in the lamina propria, close to granulocytes (Figure 1C,D), or even in intracellular vacuoles of granulocytes (Figure 1E,F). No significant alteration of the intestinal epithelial barrier was observed following incubation with the non invasive strain of *S. flexneri*, BS176 (data not shown). Detection of *S. flexneri* infection foci using anti-*S. flexneri* 5a antibody showed the presence of sub-epithelial foci in tissues cultured with M90T but no intraepithelial bacteria (data not shown). No sub-epithelial foci of bacteria was visualized in tissues cultured with BS176 or in control tissues (data not shown).

**Figure 2:**
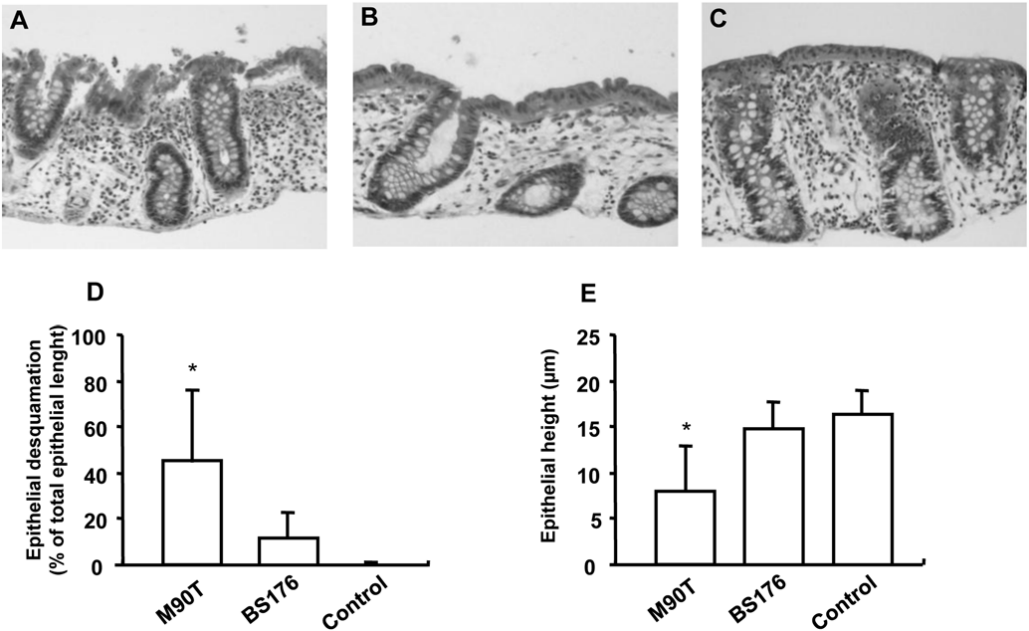
Effects of *S. flexneri* infection on the epithelium morphology of the human colon assessed by optical microscopy. (A) Infection of the colonic specimen for 3 h with the invasive strain of *S. flexneri* (M90T) induced significant desquamation of the surface epithelium. (B) In tissue infected for 3 h with the non-invasive strain (BS176), only slight desquamation could be observed. (C) No surface epithelium desquamation was observed in control non-infected tissues cultured for 3 h. (D) Quantitative analysis of colonic morphological alterations revealed that M90T induced a significant desquamation of the surface epithelium as compared to tissues infected with BS176 and non-infected tissues. (E) In addition, M90T induced a significant reduction in the height of the surface epithelium as compared to BS176 and non infected tissues. Data are expressed as mean±SEM (n = 10, * p<0.05).

In order to further quantify these alterations, desquamation and height of epithelial lining were analyzed using haematoxylin-eosin staining (Figure 2A–C). After 3 h of incubation, M90T induced a significant increase in surface epithelium desquamation as compared to BS176 and control tissues (respectively 46±32, 9±12, 0±0%; p = 0.007 and p<0.001; n = 10) (Figure 2D). Moreover, M90T significantly reduced the epithelial height as compared to BS176 and control tissues (9±5, 14±4 and 16±4 µm; respectively, p = 0.001 and p = 0.012; n = 10) (Figure 2E).

**Figure 3:**
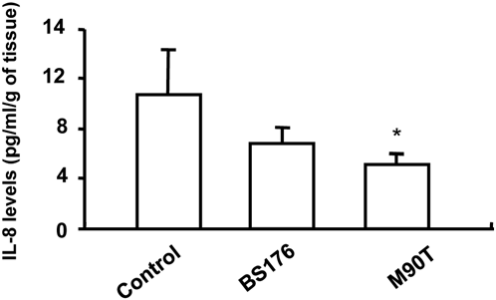
IL-8 secretion by colonic explants following infection with strains of *S.flexneri*. Following 3 h culture, IL-8 levels were significantly reduced following infection with the invasive strain (M90T) as compared to control non-infected tissue. In addition, no significant difference in IL-8 secretion following infection with the non-invasive strain (BS176) was observed as compared to control or following M90T infection. Data are expressed as mean±SEM (n = 4, * p<0.05).

At the end of the 3 h culture, surprisingly, IL-8 levels in the culture medium of control tissue was significantly larger than following incubation with M90T but not with BS176 (Figure 3).

#### 1.2 SepA is involved in morphological alterations induced by *S flexneri*

**Figure 4:**
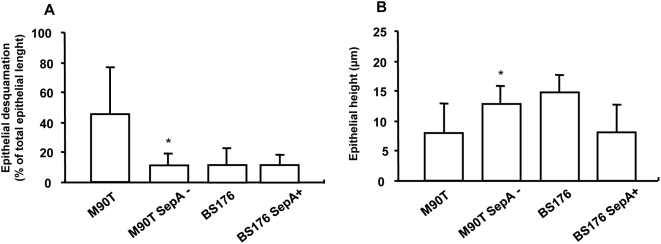
Role of SepA upon epithelial morphology in the human colon induced by *S.flexneri*. Tissues were infected for 3 h with various strains of *S.flexneri* : invasive strain (M90T), non-invasive strain (BS176), invasive strain deleted for SepA (M90T SepA−), non-invasive strain expressing SepA (BS176 SepA+). (*A*) M90T SepA− induced a significant reduction in the epithelial desquamation as compared to M90T. However, BS176 SepA+ did not increase epithelial desquamation as compared to BS176. (*B*) M90T SepA− induced a significant increase in the height of the surface epithelium as compared to M90T and BS176 SepA+ reduced the epithelial height as compared to BS176. Data are expressed as mean±SEM (n = 10, * p<0.05 as compared to M90T).

As compared with M90T, M90T SepA− infected tissues showed a significant decrease in surface epithelium desquamation (8±7 vs 46±32%; p = 0.03; n = 10) and a significantly higher epithelial height (13±3 vs 9±5 µm; p = 0.02; n = 5) (Figure 4A,B). There was no difference between BS176 SepA+ and BS176 in terms of epithelial desquamation or epithelial height (Figure 4A,B), suggesting that SepA is necessary but not sufficient to induce these alterations.

### 2 Early effects of *S. flexneri* upon enteric neurons

#### 2.1 Morphological alterations suggestive of neuronal damages

**Figure 5:**
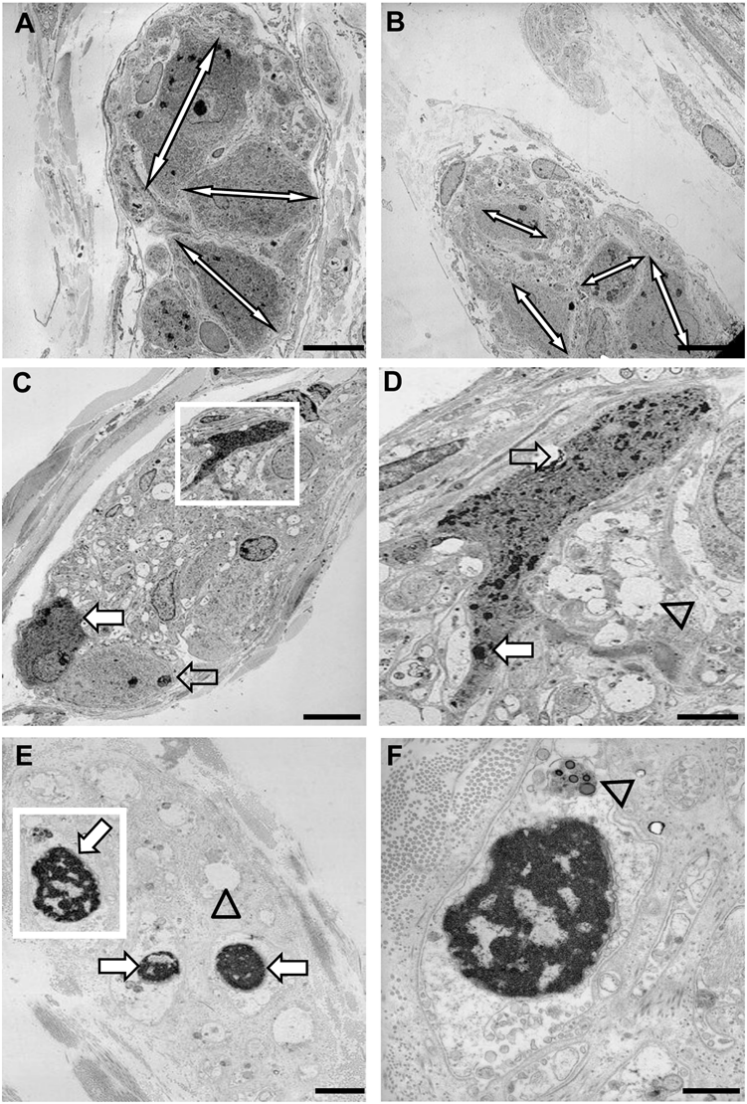
Transmission electronic microscopy of submucosal ganglia from human colonic explants infected or not with the invasive strain of *S. flexneri* (M90T). (A) Submucosal ganglia from non-infected control tissues showed neurons with normal size and shape (double arrows indicating maximal cellular diameter). (B) In contrast, submucosal ganglia from tissues infected with M90T for 3 h showed neuronal cell shrinkage. (C) Submucosal ganglia containing neurons with various stages of cell shrinkage: intact neuron (empty arrow), neuron with beginning cell shrinkage (filled arrow), neuron with advanced cell shrinkage (white square). (D) Magnified view of the selected area in C showing the presence of multilamellar bodies (empty arrow), the presence of lipofuscin granula (filled arrow) and also swollen neuronal processes (arrowhead). (E) Nerve fiber bundle observed in tissue infected with M90T showing axonal swelling (arrowhead) and also the presence of pycnotic enteric glial cell nucleus (arrow). (F) Magnified view of the selected area in E showing condensed heterochromatine of cell nucleus and the presence of lysosomatic vesicle (arrowhead) in an enteric glial cell. Bar: 10 µm (A, B, C), 2 µm (D, E), 1 µm (F).

**Figure 6:**
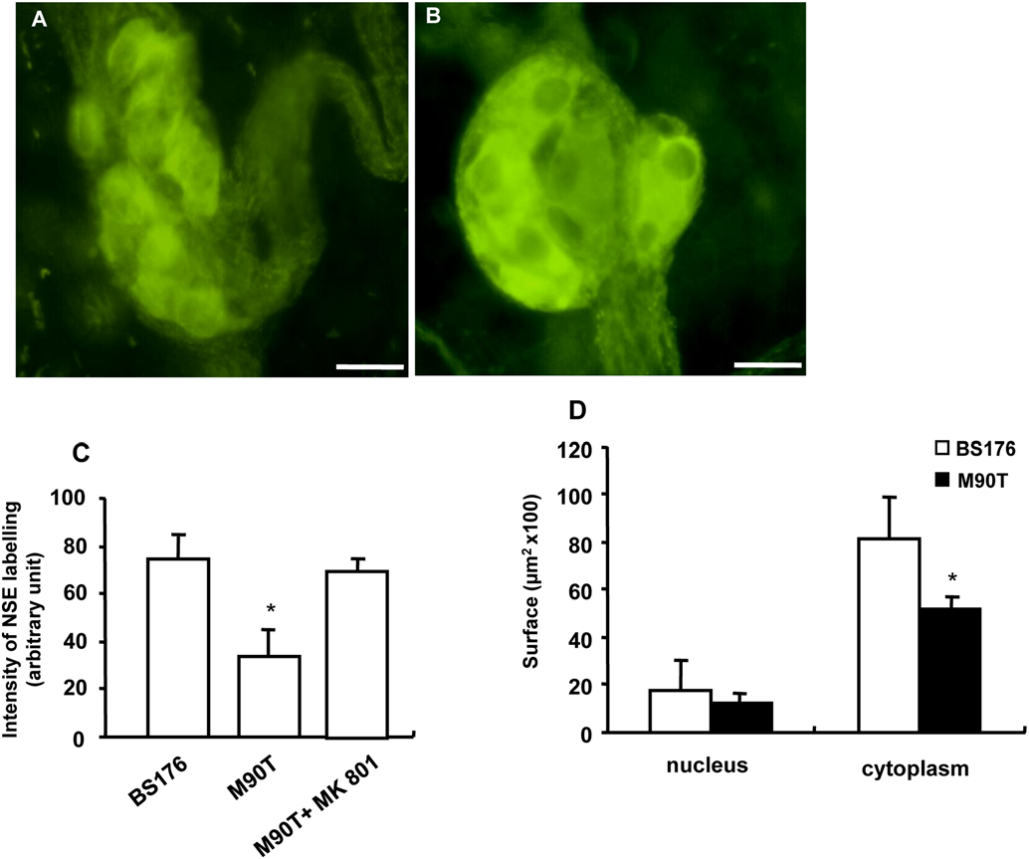
Effects of *S. flexneri* upon morphology of submucosal neurons. (A) Submucosal neurons identified with NSE showed altered morphology following 3 h infection with the invasive strain of *S. flexneri* (M90T) as compared to the non-invasive strain (BS176) (B). (C) In addition, quantitative analysis revealed that M90T induced a decrease in intensity of NSE labelling as compared to BS176 (n = 8, p<0.001). Interestingly, this decrease in intensity was prevented following pre-incubation of the NMDA receptor antagonist, MK-801 (50 µM). (D) Furthermore, M90T decreased the size of the cytoplasm but not of the nucleus in submucosal neurons as compared to BS176 (n = 6, *p = 0.002). Bar: 25 µm.

In order to determine whether M90T is able to induce degenerative changes in submucosal neurons, we performed quantitative and qualitative histological and immunohistochemical studies. TEM showed that M90T induced degenerative changes in submucosal neurons and nerve fibers such as cell shrinkage, axonal swelling, multilamellar cytoplasmic bodies and chromatin alterations of nuclei, as compared to control tissues (Figure 5A–D). Degenerative changes such as pycnotic cell nucleus were also observed in enteric glial cells (Figure 5E–F). Immunohistochemical studies showed that M90T also induced a significant decrease in the intensity of NSE labelling of enteric neurons (−45±11%; n = 8; p<0.001) as compared to BS176 (Figure 6 A–C). M90T altered also neuronal morphology, decreasing the size and in particular the cytoplasm of neurons but not of the nuclei as compared with BS176 (Figure 6D). However, the total number of neurons per ganglia identified with NSE was similar in tissues infected with M90T and BS176 (6±2 vs 7±1; n = 8; p = 0.31). Interestingly, the significant decrease in NSE labeling of enteric neurons and altered neuronal morphology induced by M90T was blocked by MK-801 (Figure 6C).

#### 2.2 Alteration of expression of enteric neuromediators

**Figure 7:**
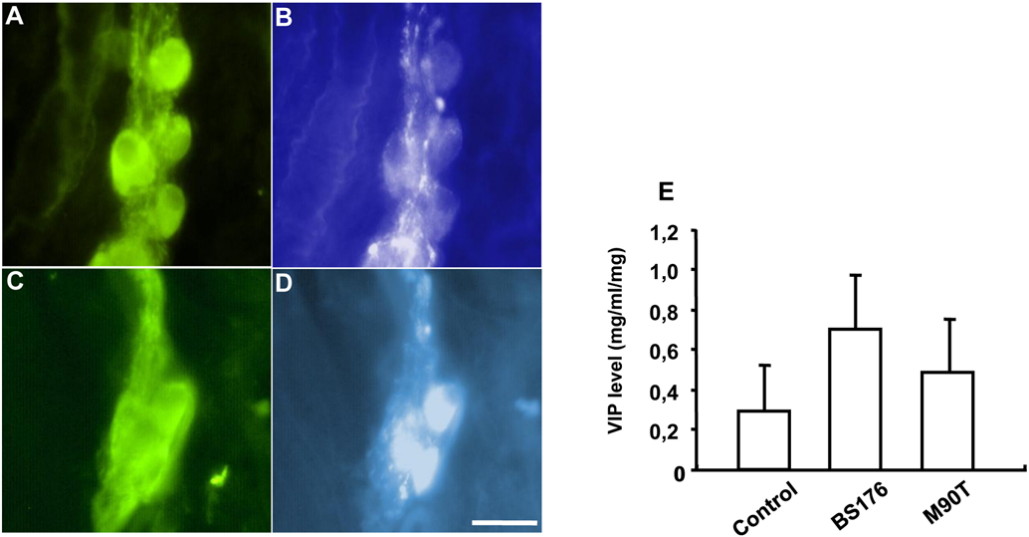
Effects of *S. flexneri* on the expression of vasoactive intestinal peptide (VIP) in submucosal neurones. In tissues infected with the non-invasive strain of *S. flexneri* (BS176), some NSE-immunoreactive (IR) neurons (A) were faintly VIP-IR (B). In contrast, following M90T infection, NSE-IR neurons (C) were strongly VIP-IR (D). Analysis of VIP content in submucosal preparation containing submucosal plexus revealed no difference in VIP content between tissue infected with M90T, BS176 or non-infected control tissue (n = 11). Bar: 25 µm.

In preparations incubated with BS176, immunohistochemical analysis of submucosal neurons showed that 29.5±6.8% of NSE-immunoreactive (IR) neurons were VIP-IR. Following incubation with BS176, the proportion of VIP-IR submucosal neurons was similar to that observed in control (non-infected) tissues (34.5±6.1%). In contrast, a significant increase in the proportion of VIP-IR neurons (49.7±4.1% of NSE-IR; p<0.001; n = 11) was observed following incubation with M90T in comparison to incubation with BS176 (Figure 7 A–D). In contrast, measurement of VIP level by ELISA in the submucosa containing the submucosal plexus revealed that VIP levels were similar in control condition compared to the condition following incubation with M90T or BS176 (Figure 7E). Addition of MK-801 did not prevent the increase in the proportion of VIP-IR neurons induced by M90T, as compared to control (data not shown).

## Discussion

In this mainly descriptive study, we sought to analyze the early histological effects of *S. flexneri* infection upon human colonic tissues, with a specific focus upon the intestinal epithelial barrier (IEB) and the ENS. Using an *ex vivo* human organ culture model, we showed that the invasive but not the non invasive strain of *S. flexneri* induced significant desquamation of the IEB which was significantly reduced following infection with SepA deficient *S. flexneri* strains. In addition, *S. flexneri* also induced rapid neuronal morphological alterations suggestive of cell death. These alterations were associated with a significant increase in the proportion of VIP-IR neurons. Morphological changes induced by *S. flexneri* but not the increase in VIP-IR were blocked by the NMDA receptor antagonist MK-801.

Various animal models have been developed aimed at better understanding the pathophysiology of shigellosis, including guinea pigs, rabbits, mice or macaques. However, higher primates and humans are the only natural hosts for *S. flexneri*. Interspecies differences in terms of immune response or ENS phenotype can therefore limit the extrapolation of animal studies to humans. In addition, the absence of data describing early effects of shigellosis in humans is mainly due to the lack or scarse material from patients infected with *S. flexneri* prior to the development of their disease and symptoms. In this context, our model has the advantage to allow the characterization of early events following *S. flexneri* infection in the human colon upon both the IEB and the ENS. It also presents the advantage over reductionist cellular models to integrate various components of the mucosa such as IEC, submucosal neurons and resident inflammatory cells.

One major finding of the study was the presence of a massive desquamation of surface epithelium induced by *S. flexneri*, as early as three hours post-infection. These results are reminiscent of observations obtained *in vivo*, both in animal models and in patients with shigellosis [7], [8]. In particular, human intestinal segments grafted into SCID mice infected with *S. flexneri* showed focal desquamation of the epithelial lining without ulcerations, similar to our observations [8]. Interestingly, the lesions of the mucosa were not reduced in polynuclear deficient mice [8]. This is consistent with our observations since no recruitment of neutrophils can occur in the colonic explants used in our experiments. In addition, damages of the mucosa occurred in area devoid of M cells suggesting that, in our model, *S. flexneri* does not require entry through Peyer's patches. A striking finding was the rapidity of the lesions induced by *S. flexneri* which occurred within 3 h post-infection. This could be due to the high inoculum of bacteria used and the absence of peristaltism and secretory response in our *ex vivo* model. In addition, a basal inflammation in the explant, probably due to hypoxic conditions generated by the culture and absence of vascularisation, might also have potentiated the mucosal alterations induced by *S. flexneri* [9]. Interestingly, no bacteria was identified within intestinal epithelial cells, although they were present in some immune cells, but mainly directly beneath the shedding epithelial layer. This observation is also consistent with *in vivo* animal studies showing few bacteria in the epithelium but localized mainly in the lamina propria and submucosa [7]–[9].

Another important finding of this study is the demonstration that SepA plays a key role in the generation of mucosal damages induced by *S. flexneri*. Indeed, invasive bacteria deficient for SepA significantly reduced epithelial damage. Our results extend to human tissue the role of SepA described in rabbit, where SepA deficient *S. flexneri* significantly reduced the ability of *S. flexneri* to induce mucosal atrophy and tissue inflammation [11]. The mechanisms of action of SepA, a serine protease, remain unknown although studies have suggested that proteases via their activation of protease activated receptor are involved in the pathogenesis of GI inflammation, in part by increasing paracellular permeability [18]–[20]. Interestingly, an increase in paracellular permeability is observed in human mouse xenograft model prior to any mucosal damage [8] but the involvement of SepA in these effects remain unknown.

One surprising and unexpected finding of this study was the decrease in IL-8 induced by *S. flexneri* in our model. This result can probably be explained in part by recent findings showing that *S. flexneri* produces various anti-inflammatory proteins, which dampen the inflammatory response induced by the bacteria. Indeed, proteins encoded and secreted by *S. flexneri* such as OspG or OspF prevent nuclear translocation of NF-κB or impair the accessibility of NF-κB to promoters including IL-8, respectively [21]. The action of these proteins could be unraveled in our model as non-infected colonic tissue secretes high level of IL-8, probably due to the hypoxia of the explants, as previously reported [22]. The decrease of IL-8 secretion induced by *S. flexneri* could also be due to the death of IL-8 secreting cells such as IEC, immune cells or even neurons [17].

Finally we showed that *S. flexneri* has the potential to induce degenerative processes both in enteric neurons and glial cells. Until recently, only mucosal components of the gut such as epithelial cells, immune cells were sought to be the target of *S. flexneri*. Invasion of neurons by *S. flexneri* is probably not necessary to induce neuronal damages as 1) no bacteria were detected both by means of transmission electronic microscopy and immunohistochemical methods, 2) the effects are significantly reduced with an NMDA receptor antagonist. This latter suggest that excitotoxic effects of glutamate could be involved in neurodegenerative processes induced following infection with *S. flexneri*. A previous study has shown that glutamate, via the activation of NMDA receptors, can induce cell death in enteric neurons *in vivo* or *ex vivo* [23]. The origin of the glutamate responsible for these effects is currently unknown but could be produced by *S. flexneri* itself, cells of the mucosal environment or even neurons. The increase in the proportion of VIP-IR neurons observed in this study is probably more to be associated with neurodegenerative processes than to changes in neuronal phenotype. Indeed, the total VIP level in the submucosal plexus was not altered following incubation with *S. flexneri*, as compared to controls. Therefore, the increase in the proportion of VIP-IR enteric neurons is probably due to reduced axonal transport of VIP and its subsequent accumulation in the neuronal cell body. Indeed, axonal transport is significantly affected and reduced during early neurodegenerative processes [24].

In conclusion, using an *ex vivo* model, we have shown that infection by *S. flexneri* induces rapid mucosal and neuronal alterations in the human colon. In particular, we have shown the major role of SepA in the induction of mucosal desquamation while NMDA dependent pathways could account for *S. flexneri* – induced degenerative processes in the ENS. Finally, this human model should allow us to gain better insight into the early pathogenic events following *S. flexneri* infection and the mechanisms involved.
